# Interest In and Practices Related to Gynecologic Oncology among Members of the Brazilian Federation of Associations of Gynecology and Obstetrics

**DOI:** 10.1055/s-0039-1692467

**Published:** 2019-06

**Authors:** Arilto Eleutério da Silva Júnior, Jesus Paula Carvalho, Sophie Françoise Mauricette Derchain, Angélica Nogueira Rodrigues, Renato Moretti, Eduardo Batista Cândido, Ricardo dos Reis, Aline Evangelista Santiago, Agnaldo Lopes da Silva Filho

**Affiliations:** 1 Department of Obstetrics and Gynecology, Faculdade de Medicina de Botucatu, Universidade Estadual Paulista “Júlio de Mesquita Filho,” Botucatu, SP, Brazil; 2 Department of Obstetrics, Faculdade de Medicina, Universidade de São Paulo, São Paulo, SP, Brazil; 3 Department of Obstetrics and Gynecology, Faculdade de Medicina, Universidade Estadual de Campinas, São Paulo, SP, Brazil; 4 Department of Gynecology, Faculdade de Medicina, Universidade Federal de Minas Gerais, Belo Horizonte, MG, Brazil; 5 Center of Oncology, Hospital Israelita Albert Einstein, São Paulo, SP, Brazil; 6 Department of Oncology Gynecology, Hospital de Amor de Barretos, Barretos, SP, Brazil

**Keywords:** gynecology, women – diseases, oncology, cancer – surgery, gynecologists, obstetricians, ginecologia, mulheres – doenças, oncologia, câncer – cirurgia, ginecologistas, obstetras

## Abstract

**Objective** The present study aims to obtain basic demographic information, the level of interest and of training in gynecology oncology among Brazilian obstetricians and gynecologists (OB-GYNs) to create a professional profile.

**Methods** An online questionnaire was sent to 16,008 gynecologists affiliated to the Brazilian Federation of Associations of Gynecology and Obstetrics (FEBRASGO, in the Portuguese acronym). We considered gynecologists dedicated to gynecologic oncology (OB-GYNs ONCO) those who self-reported that > 50% of their daily practice consists in working with women's cancer care.

**Results** A total of 1,608 (10%) of 16,008 FEBRASGO members responded. The OB-GYNs are concentrated in the southern and southeastern states of Brazil. Gynecologic oncology was considered the 8^th^ greatest area of interest in gynecology among the OB-GYNs. A total of 95 (5.9%) of the OB-GYNs were considered OB-GYNs ONCO. Obstetricians and gynecologists are actively engaged in cancer care: > 60% of them dedicate up to 25% of their daily practice to oncology. The role of the physicians in screening and prevention, diagnosis, in the treatment of precancerous lesions, and in low complexity surgical procedures is notably high. Gynecologists dedicated to gynecologic oncology in Brazil have a heterogeneous, nonstandardized and short training period in gynecologic oncology. These professionals had a more significantly role in performing medium- and high-complexity operations compared with OB-GYNs (65.2% versus 34%, and 47.3% versus 8.4%, respectively).

**Conclusion** The role of OB-GYNs and of OB-GYNs ONCO appears to be complementary. Obstetricians and gynecologists act more often in screening and prevention and in low-complexity surgical procedures, whereas OB-GYNs ONCO are more involved in highly complex cases. Strategies to raise standards in cancer training and to encourage the recognition of gynecologic oncology as a subspecialty should be adopted in Brazil.

## Abstract (pt)

**Resumo:**

**Objetivo** O presente estudo tem como objetivo obter informações demográficas básicas, o nível de interesse e de treinamento em ginecologia oncológica entre obstetras e ginecologistas (OB-GYNs) brasileiros para criar um perfil destes profissionais.

**Métodos** Um questionário online foi enviado a 16.008 ginecologistas filiados à Federação Brasileira de Associações de Ginecologia e Obstetrícia (FEBRASGO). Nós consideramos ginecologistas dedicados à oncologia ginecológica (OB-GYNs ONCO) aqueles que referiram atuar em > 50% de sua prática diária com o tratamento do câncer feminino.

**Resultados** Um total de 1.608 (10%) dos 16.008 membros da FEBRASGO responderam ao questionário. Os OB-GYNs estão concentrados nos estados do sul e sudeste do Brasil. A oncologia ginecológica foi considerada a 8ª área de maior interesse em ginecologia entre os OB-GYNs. Um total de 95 (5,9%) dos OB-GYNs foram considerados ginecologistas dedicados à oncologia ginecológica (OB-GYNs ONCO). Obstetras e ginecologistas estão ativamente envolvidos no tratamento do câncer: > 60% deles dedicam até 25% de sua prática diária à oncologia. O papel dos médicos na triagem e na prevenção, no diagnóstico, no tratamento de lesões pré-cancerosas e em procedimentos cirúrgicos de baixa complexidade é notavelmente alto. Ginecologistas dedicados à oncologia ginecológica no Brasil têm um período de treinamento em oncologia ginecológica heterogêneo, não padronizado e curto. Estes profissionais tiveram um papel mais significativo na realização de operações de média e alta complexidade em comparação com OB-GYNs (65,2% *versus* 34%, e 47,3% *versus* 8,4%, respectivamente).

**Conclusão** Os papéis dos OB-GYN e dos OB-GYNs ONCO parecem ser complementares. Os OB-GYNs frequentemente atuam em triagem e prevenção e em procedimentos cirúrgicos de baixa complexidade, enquanto os OB-GYNs ONCO estão mais envolvidos em casos de mais alta complexidade. Estratégias para elevar os padrões de treinamento em oncoginecologia e incentivar o reconhecimento da oncologia ginecológica como uma subespecialidade devem ser adotadas no Brasil.

## Introduction

It is estimated that the occurrence of gynecological cancers in Brazil is of ∼ 30,000 new cases per year. For 2018, 16,370 new cases of cervical cancer, 6,600 new cases of uterus cancer, and 6,150 new cases of ovarian tumors are expected.^1^ Prevention, early diagnosis, and treatment in the right time are fundamentals of optimal cancer care.^2^ In low- and middle-income countries, investments in preventive measures are more cost-effective for cancer control. One-third to one-half of all cancer deaths could be avoided through prevention and early detection and treatment.^3^

There are ∼ 28,280 obstetricians and gynecologists (OB-GYNs) in Brazil, and 16,008 of them are affiliated to the Brazilian Federation of Associations of Gynecology and Obstetrics (FEBRASGO, in the Portuguese acronym [https://www.febrasgo.org.br/]). Obstetricians and gynecologists may act as primary care physicians and, occasionally, are the only physicians of the women.^4^ Obstetricians and gynecologists are integral in treatments for gynecological cancer, which comprise preventive measures, screening, diagnosis, and treatment, ranging from low- to high-complexity cases.^5^ Obstetricians and gynecologists usually receive some training in gynecologic oncology during the medical residence.

It is well established that outcomes of gynecologic cancer patients are better when treated by appropriately trained gynecologic oncologists (OB-GYNs ONCO).^6^ ^7^ ^8^ ^9^ ^10^ However, this subspecialty is not recognized in Brazil, and general surgeons, oncology surgeons, and OB-GYNs are currently the specialists responsible for gynecologic cancer surgeries. Neither surgical nor gynecologic societies can certify professionals who dedicate themselves to gynecologic cancer treatment, hampering gynecologic oncology training in the country.^11^

There is no data regarding who are the OB-GYNs involved in women's cancer care in Brazil or those who are more dedicated to gynecologic oncology. Therefore, the present study aims to obtain basic demographic information, the level of interest and of training in gynecologic oncology among OB-GYNs. A profile of these professionals may help in the improvement of the oncological attention in our country.

## Methods

After obtaining approval from the Institutional Review Board (number 2.447.492) and from the Brazilian FEBRASGO, we obtained a full mailing list of all of the FEBRASGO members. A questionnaire was developed to collect demographic data from Brazilian OB-GYNs of all of the regions of the country.

The emails were sent to 16,008 OB-GYNs on October 19, 2016. The questionnaire was made available through a free survey Web site (SurveyMonkey [SurveyMonkey, San Mateo, CA, USA]), and the answers were received via internet between October 19, 2016 and November 21, 2016. The survey included 28 separate questions, and it took ∼ 15 minutes to complete. The respondents were asked about demographic characteristics, including their current practice setting, personal training history, experience with minimally invasive procedures, and their ability to perform complex surgical procedures. The collected data were kept confidential, and the identities of the interviewees were omitted and preserved anonymously. All of the collected data was stored by the FEBRASGO and shared with the authors.

We considered OB-GYNs ONCO those OB-GYNs who self-reported that > 50% of their daily practice consisted in working with women's cancer care. The present study aimed to evaluate the clinical practice in gynecologic oncology procedures; therefore, diagnostic laparoscopy and hysteroscopy were considered low-complexity procedures. Medium-complexity procedures included unilateral/bilateral salpingo-oophorectomy, unilateral/bilateral ovarian cystectomy, and hysterectomy with or without unilateral/bilateral salpingo-oophorectomy. High-complexity procedures included radical hysterectomy, pelvic and/or para-aortic lymphadenectomy, splenectomy, small bowel/colon resection, peritonectomy, hepatectomy, and diaphragm stripping.^12^

### Statistical Analysis

The data collected were analyzed using frequency distributions tests; all of the unknown or missing responses were removed from the analysis. All of the statistical analyses were performed using IBM SPSS Statistics for Windows, Version 22.0 (IBM Corp., Armonk, NY, USA). *P-values* < 0.05 were considered statistically significant.

## Results

There were 1,608 OB-GYNs willing to respond to the questionnaire; 10% of FEBRASGO members answered the questions. A fully completed questionnaire was sent back by 1,272 (70%) OB-GYNs. All of the answers received were included in the present analysis.

**Fig. 1:**
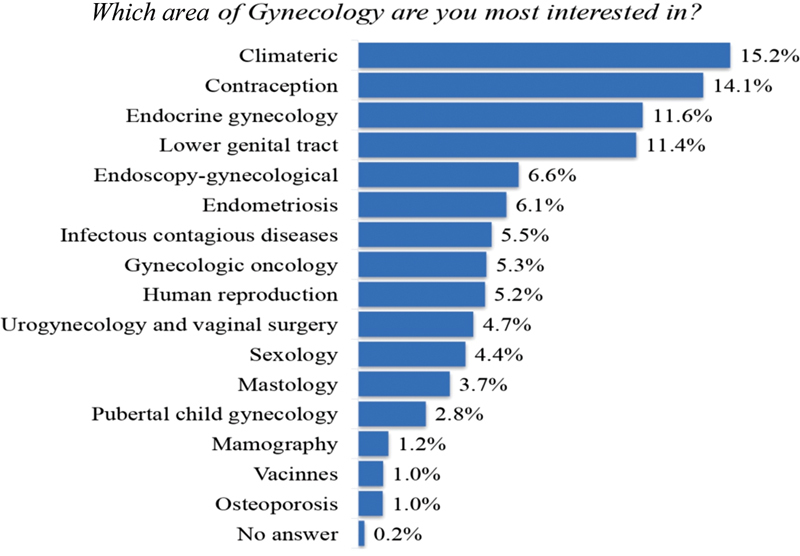
Major areas of interest in gynecology among obstetricians and gynecologysts in Brazil (1,606 respondents).

**Fig. 2:**
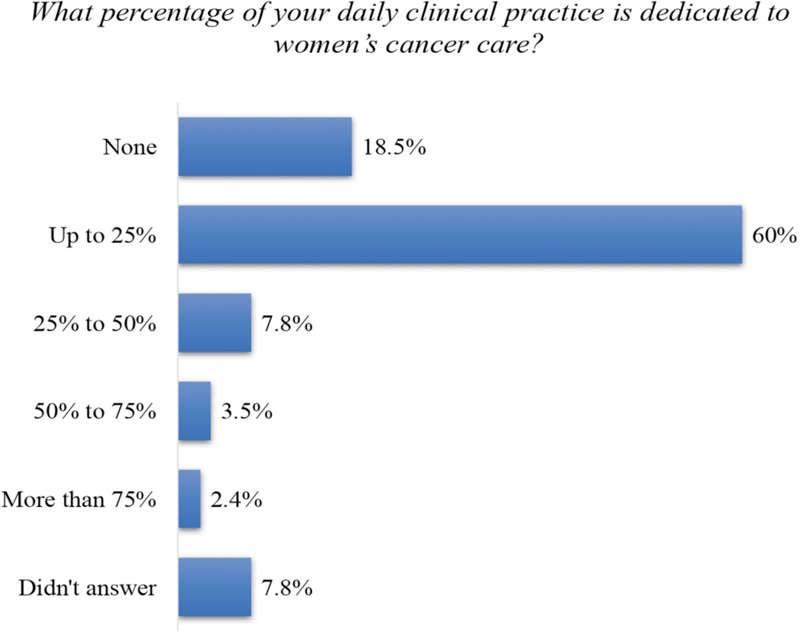
Percentage of the daily clinical practice dedicated to women's cancer care among obstetricians and gynecologysts in Brazil(1,493 respondents).

Gynecology oncology was considered only the 8^th^ greatest area of interest in gynecology among the OB-GYNs (Fig. 1). In reporting the percentage of daily clinical practice dedicated to women's cancer care, 78.5% of the respondents did not treat any case of cancer or did it in < 25% of their daily practice in the previous 12 months (Fig. 2). More than 60% of the OB-GYNs dedicated up to 25% of their daily practice to oncology. A total of 95 (5.9%) of the OB-GYNs self-reported that > 50% of their daily practice consisted in working with women's cancer care and were considered OB-GYNs ONCO.

**Fig. 3:**
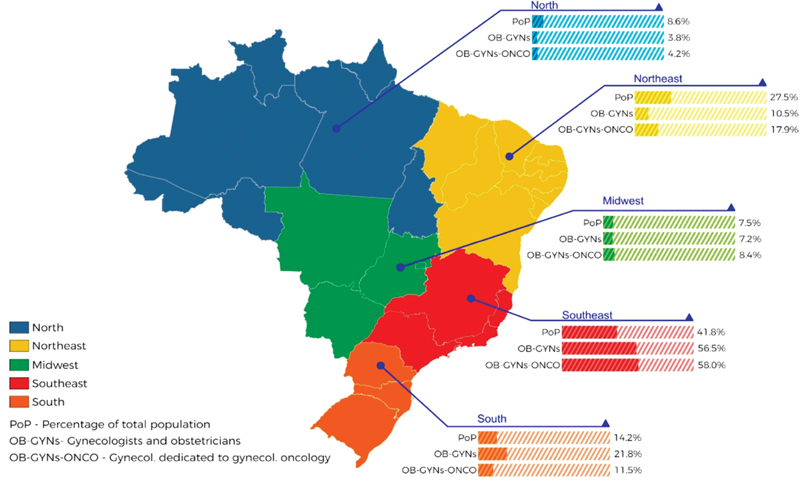
Distribution of the obstetricians and gynecologysts in different regions of Brazil. (1,608 respondents). **Note:** OB-GYNs: obstetricians and gynecologists. OB-GYNs ONCO: OB-GYNs who self-reported that > than 50% of their daily practice consists in working with women's cancer. (Distribution of OB-GYNs ONCO by region: South: 11; Southeast: 55; North: 4; Midwest: 8; Northeast: 17).

As illustrated in Fig. 3, both OB-GYNs and OB-GYNs ONCO are concentrated in the southern and southeastern states of the country. These professionals are also concentrated in larger cities; 42.8% of the OB-GYNs and 54.7% of the OB-GYNs ONCO work in cities with > 1 million inhabitants. The majority of the OB-GYNs and OB-GYNs ONCO were board-certified by FEBRASGO in gynecology and obstetrics (66% and 68.4%, respectively).

Regarding the gynecologic oncology training of OB-GYNs ONCO, 76.8% had performed a residence program, and 60% of them had specialization courses in gynecology oncology. A total of 29 (30.5%) of the OB-GYNs ONCO have master's, doctoral and/or postdoctoral degrees. Finally, 11.5% had been trained in a foreign country in gynecologic oncology for at least 3 months. The length of training in gynecology oncology was ≥ 24 months in 49.4% of the OB-GYNs ONCO. Less than half (48.5%) of the OB-GYNs ONCO performed minimally invasive surgeries. Only 17.9% of them performed laparoscopy or robotics in > 30% of the surgical cases. A high percentage of the OB-GYNs ONCO (76.8%) are affiliated to at least one Gynecology Oncology Society, such as the Society of Gynecologic Oncology (SGO), the European Society of Gynecological Oncology (ESGO), and the International Gynecologic Cancer Society (IGCS).

**Fig. 4:**
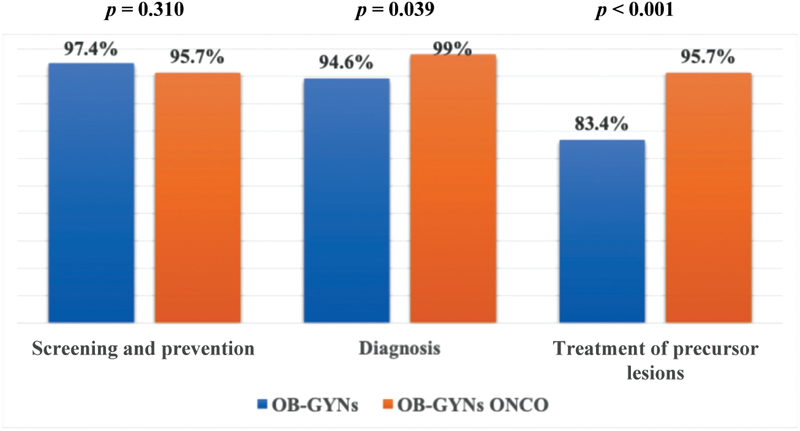
Comparison between OB-GYNs and OB-GYNs ONCO regarding their role in screening/prevention, diagnosis, and treatment of precursor lesions. **Note:** OB-GYNs: obstetricians and gynecologists. OB-GYNs ONCO: OB-GYNs who self-reported that > 50% of their daily practice consists in working with women's cancer. Differences between groups were calculated using the chi-squared test (1,598 respondents).

**Fig. 5:**
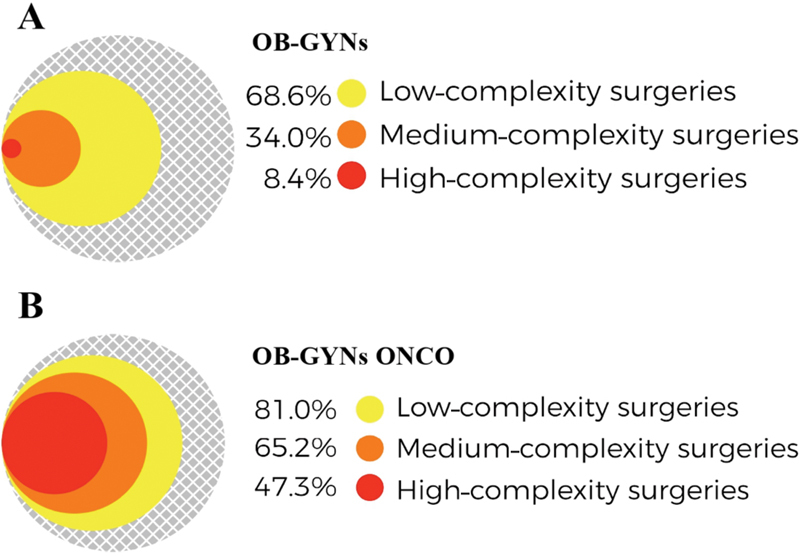
Comparison between OB-GYNs (A) and OB-GYNs ONCO (B) regarding the complexity of oncological surgeries performed in the clinical practice. **Note:** OB-GYNs: obstetricians and gynecologists. OB-GYNs ONCO: OB-GYNs who self-reported that > 50% of their daily practice consists in working with women's cancer (1,558 respondents).

A large percentage (45.2%) of the OB-GYNs ONCO still practices obstetrics. Only 46.3% of these physicians had > 30 gynecological cancer surgeries per year, and 25% of them had between 6 and 30 cases in the previous 12 months. Only 47.3% of them self-reported that they were able to perform high complexity procedures. Figs. 4 and 5 illustrate the comparison between the roles of OB-GYNs and of OB-GYNs ONCO in women's cancer care. Both groups had broad action in prevention, screening, treatment of precursor lesions, and in low-complexity surgical procedures. Gynecologic oncologists had a significantly higher role in the diagnosis of cancer and in the treatment of precursor lesions compared with general OB-GYNs (*p* = 0.039 and *p* < 0.001, respectively). Regarding oncological surgeries, OB-GYNs ONCO performed more low-, medium-, and high-complexity procedures compared with general OB-GYNs (*p* < 0.001). The greatest difference between the groups was in the rate of medium- and high-complexity operations (34% *versus* 65.2%, and 8.4% *versus* 47.3%, respectively) (Fig. 5).

## Discussion

The distribution of the OB-GYNs in the five regions of Brazil shows a concentration of these professionals in southeast and south regions, and a deficit in the northeast and north regions. Another study has shown that the distribution of physicians in municipalities grouped by population strata brings a new dimension to the same problem.^13^ The 39 cities with > 500 thousand inhabitants concentrate 30% of the population and 60% of all physicians in the country. When women with gynecological cancers are treated by OB-GYNs ONCO in referral cancer centers, they are able to live longer and with a better quality of life.^6^ ^8^ ^10^ ^14^ Therefore, ideally, the patients should be referred to high-volume physicians/hospitals to increase their life expectancy and quality of life.^6^ ^7^ ^9^ The adoption of strategies for creating centers specialized in gynecologic oncology for the referral of women with cancer in Brazil should be encouraged.^9^

Paradoxically, OB-GYNs demonstrated little interest in gynecologic oncology, which was in contrast with the involvement of the board in women's cancer care. Almost 95% of the OB-GYNs are involved in prevention, screening, diagnosis and treatment, and > 60% of these physicians dedicate up to 25% of their daily practice to issues related to oncology. Obstetricians and gynecologists usually receive some training in gynecologic oncology during medical residence, but this training usually takes a only few weeks of the program, is not homogeneous, and is probably insufficient to provide adequate preparation to care for women with gynecological cancer.^9^ ^15^ ^16^ As OB-GYNs are often the initial point of contact for patients, and as they also may make referrals, a better curriculum associated with a longer and higher quality cancer prevention, screening and surgical training program during residency should be a strategy to improve the knowledge and abilities of OB-GYNs in gynecologic oncology.

According to the definition of the American Board of Obstetrics and Gynecology, an OB-GYN ONCO is “a specialist in obstetrics and gynecology who is prepared to provide consultation on comprehensive management of patients with gynecologic cancer and who works in an institutional setting wherein all the effective forms of cancer therapy are available.”^6^ The present study showed that OB-GYNs ONCO care for a low volume of cancer cases, and a large number of them continue to work with obstetrics. These facts suggest a nonintegral dedication to women's cancer care, which may reflect negatively in the treatment outcomes. It has been demonstrated that specialized physicians who work in multidisciplinary teams to treat women with gynecological cancers are able to obtain the best clinical and oncological outcomes.^6^ ^7^ ^8^ ^10^

Gynecologic oncologists have an essential role when treating women with gynecological cancer.^10^ Our study showed that the OB-GYNs ONCO in Brazil have a heterogeneous, nonstandardized, and short training period in gynecologic oncology. The nonrecognition of gynecologic oncology as a medical subspecialty represents a huge barrier to the complementary training of these professionals.^9^ In high-income countries, physicians who want to be gynecologic oncologists need to undergo a long and specific period of training and education.^6^ ^16^ ^17^ After finishing 4- to 5-year residency-training programs in obstetrics and gynecology, these professionals need more 2 to 4 years completing a specific fellowship-training program in gynecologic oncology.^6^

The training, skills, and knowledge base required of an OB-GYN ONCO are rapidly expanding.^10^ The results of the present study showed a low rate of minimally invasive surgery, and only 47.3% of the OB-GYNs ONCO were able to perform high-complexity oncological procedures. These findings suggest the absence of standardized training. Because the training of these professionals is highly diverse, one of the ways to equate quality of care is through the standardization of formal programs of specialization. These programs should be focused in gynecologic oncology with consistent qualification of professionals, such as multidisciplinary tumor boards, treatment guidelines tailored to local needs, tumor registries, clinical research, screening, and palliative programs.^6^ ^15^

The present study has several limitations. The study is based on physician self-reports of involvement in cancer care. The results may not represent the views of the entire community of OB-GYNs ONCO because clinical oncologists, general surgeons, and oncologist surgeons were not included. It is possible that the participating physicians have more cancer care involvement than physicians who were not willing to answer the survey. Recall bias may also have been present. Members may not have accurately recalled the number and the percentage of procedures performed.

However, to the best of our knowledge, the present study is the first to create a profile of the OB-GYNs involved in women's cancer care in Brazil. The role of OB-GYNs and of cancer specialists in cancer care appears to be complementary. Obstetricians and gynecologists act more often in screening, in prevention, and in low-complexity surgical procedures, whereas OB-GYNs ONCO are more involved in high-complexity cases, especially those requiring surgical treatment. Efforts should focus on the training of OB-GYNs in gynecologic oncology during medical residency and recruitment, on specialized training in gynecologic oncology, and on the use of specialists in gynecologic oncology for the treatment of high-complexity cases.^15^

## Conclusion

These strategies should be supported by policies to organize the health system, to refer high-complexity patients to specialized centers, and to invest in cancer registries, patient navigators, social workers, or other personnel to help patients coordinate their care and relieve their health-related burden. Strategies should be adopted to successfully raise standards in cancer training and to encourage the recognition of gynecologic oncology as a subspecialty in Brazil.
